# Supplementary figures and images for: Species-specific quantification of circulating ebolavirus burden using VP40-derived peptide variants
Source: PLoS Pathog. 2021 Nov 8;17(11):e1010039. doi: 10.1371/journal.ppat.1010039 (PMC8601621; doi:10.1371/journal.ppat.1010039)

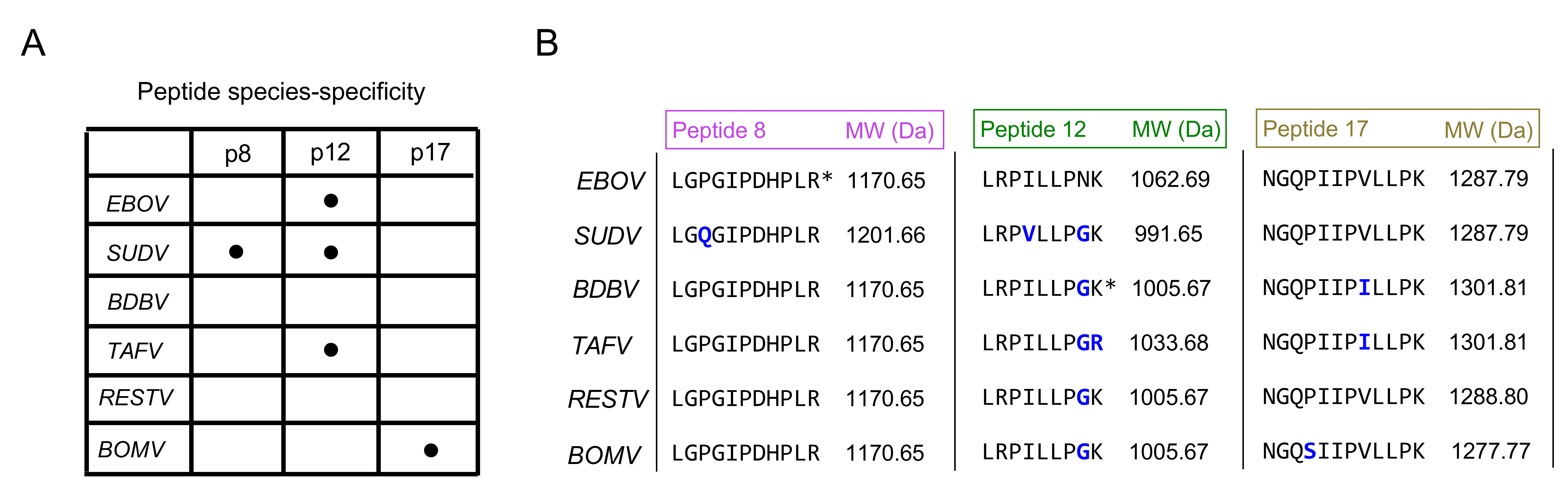

Supplement: S1 Fig — (TIF) [file ppat.1010039.s001.tif]

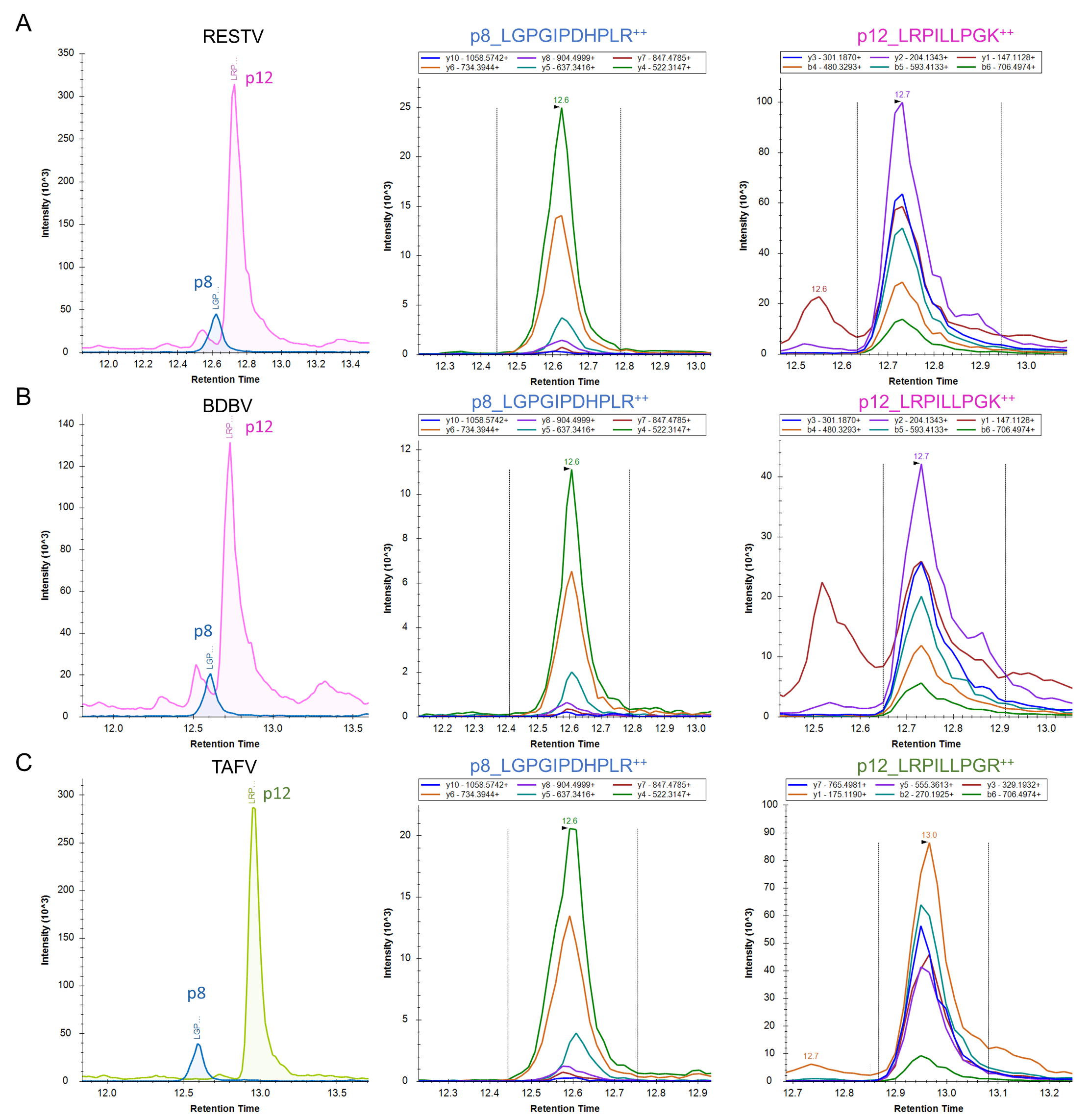

Supplement: S2 Fig — MRM analysis of two VP40 peptide variants from the PBS solution spiked with 0.2 μg/μL total viral protein of inactivated authentic virions from (A) RESTV, (B) BDBV and (C) TAFV. (TIF) [file ppat.1010039.s002.tif]

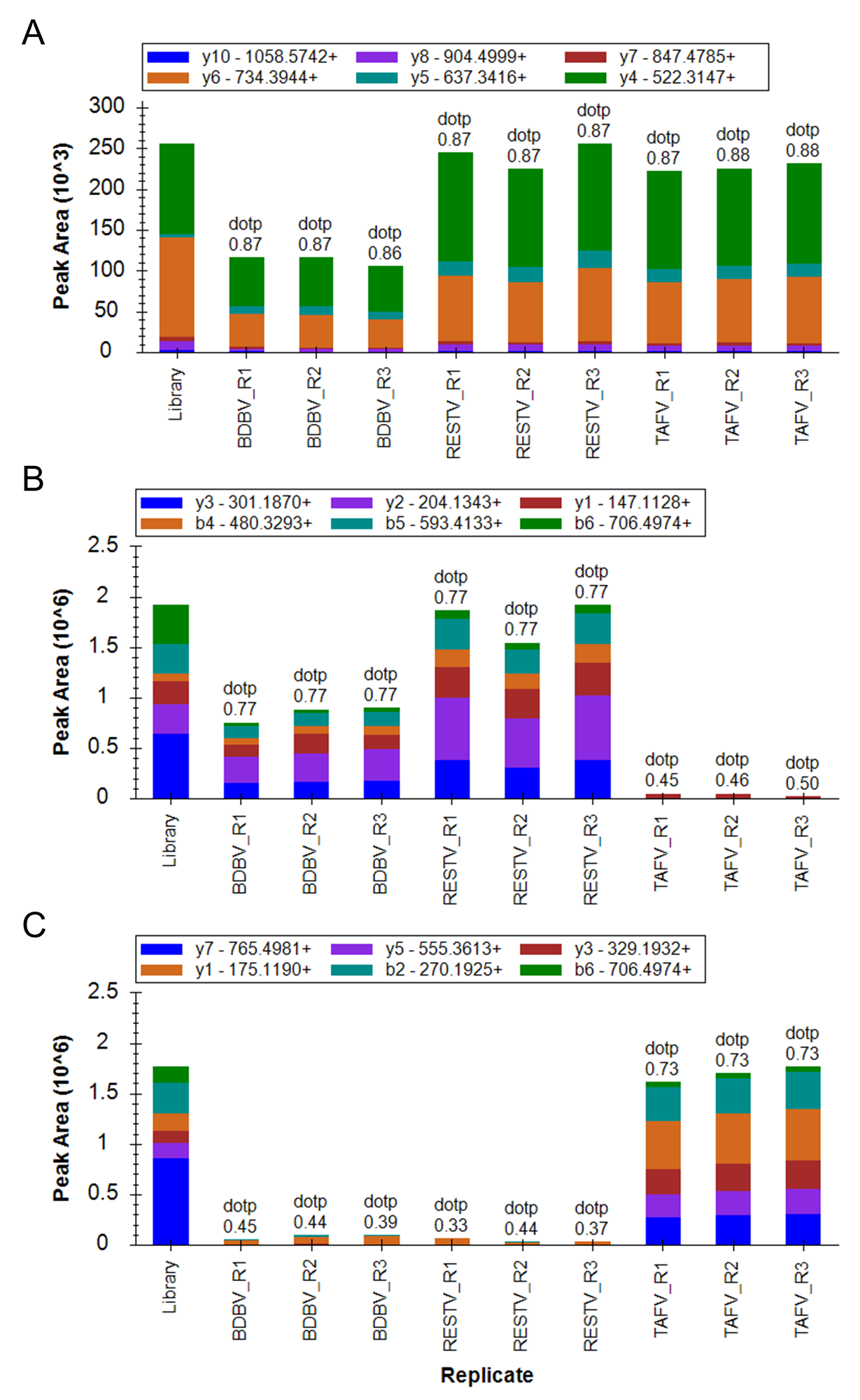

Supplement: S3 Fig — Reproducibility of extracted peak area of (A) VP40 peptide 8 with sequence LGPGIPDHPLR shared by the three species, (B) VP40 peptide 12 with sequence LRPILLPGK shared by BDBV and RESTV, and (C) VP40 peptide 12 with sequence LRPILLPGR from TAFV. (TIF) [file ppat.1010039.s003.tif]

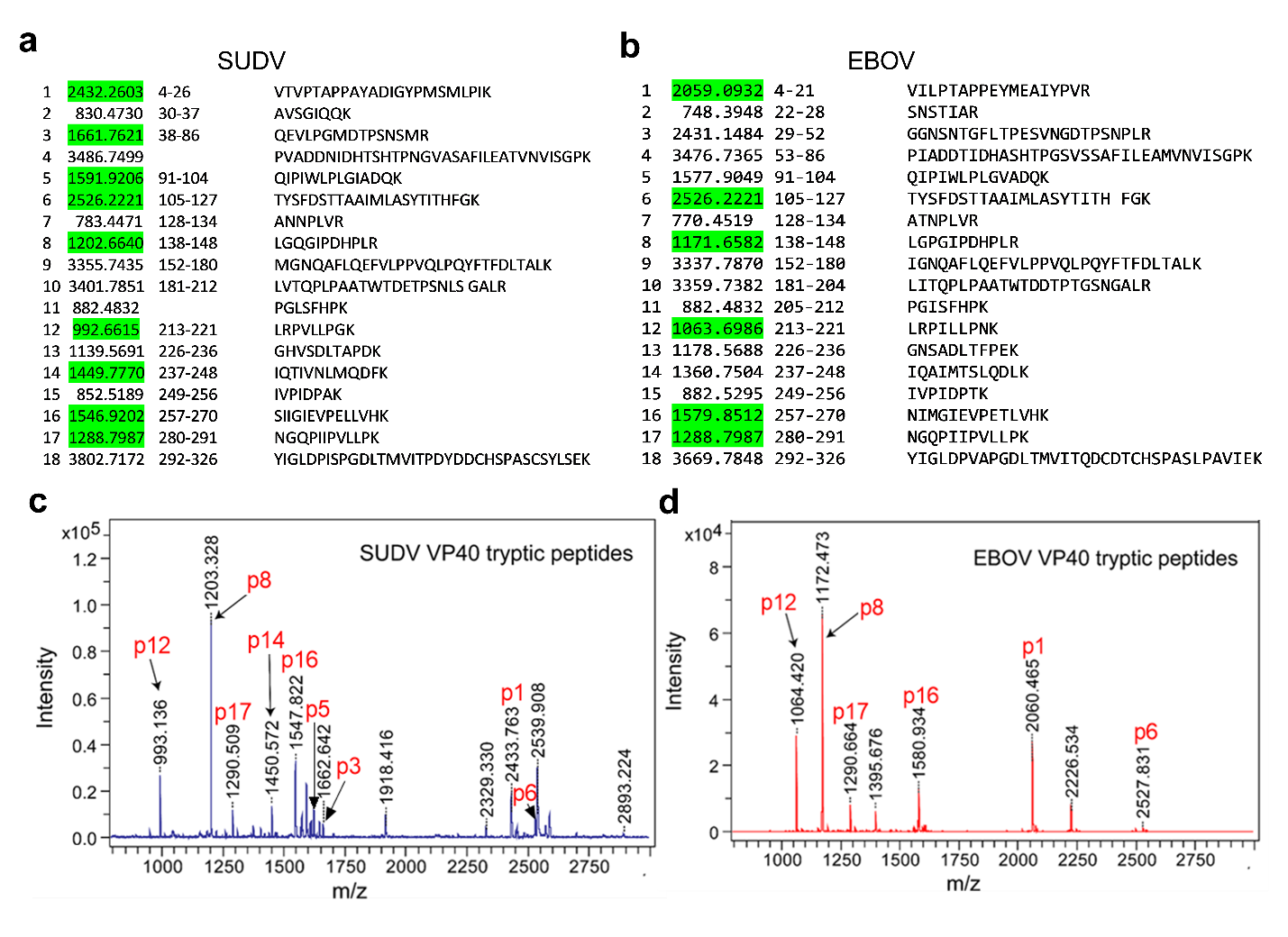

Supplement: S4 Fig — (TIF) [file ppat.1010039.s004.tif]

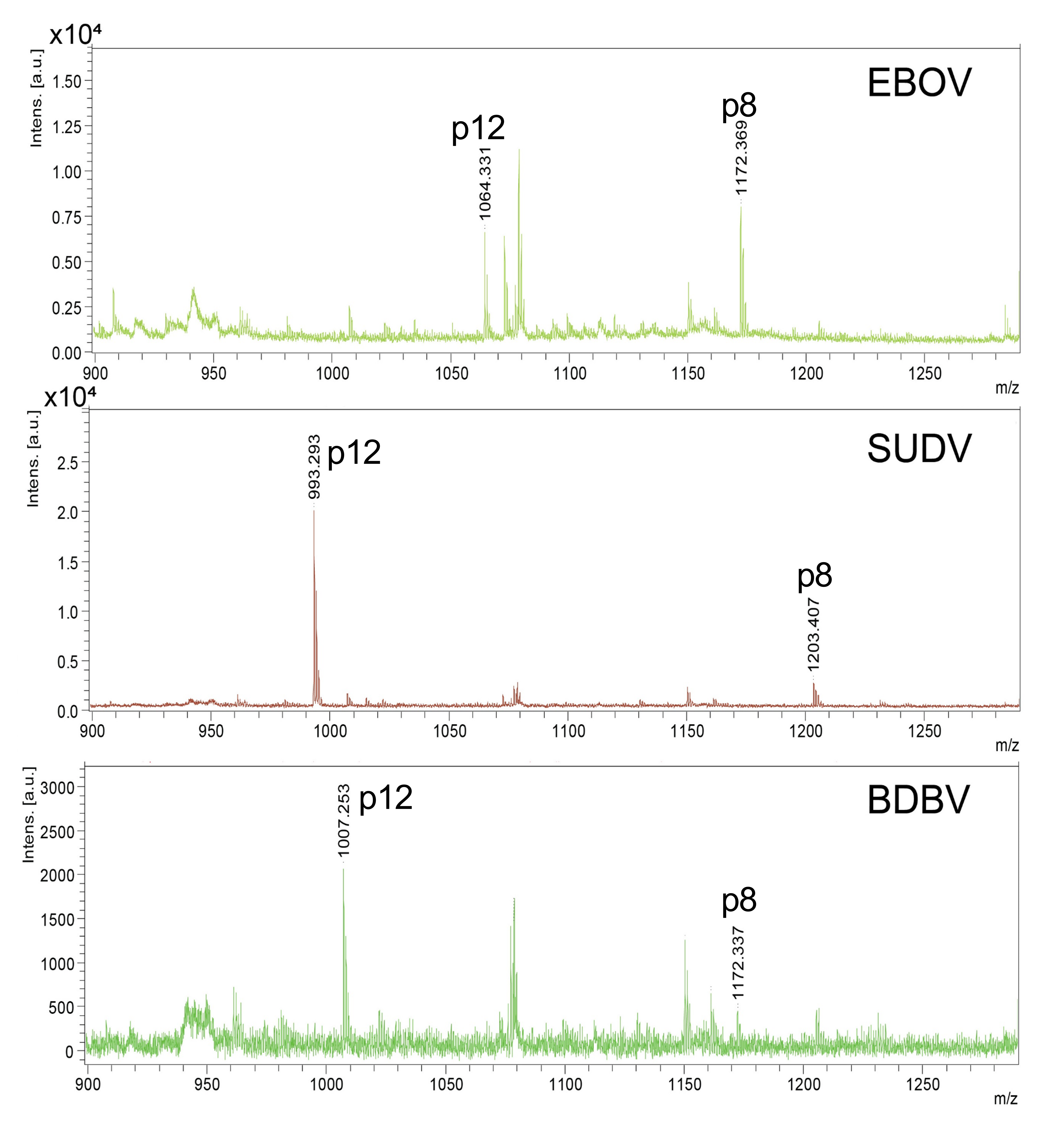

Supplement: S5 Fig — (TIF) [file ppat.1010039.s005.tif]

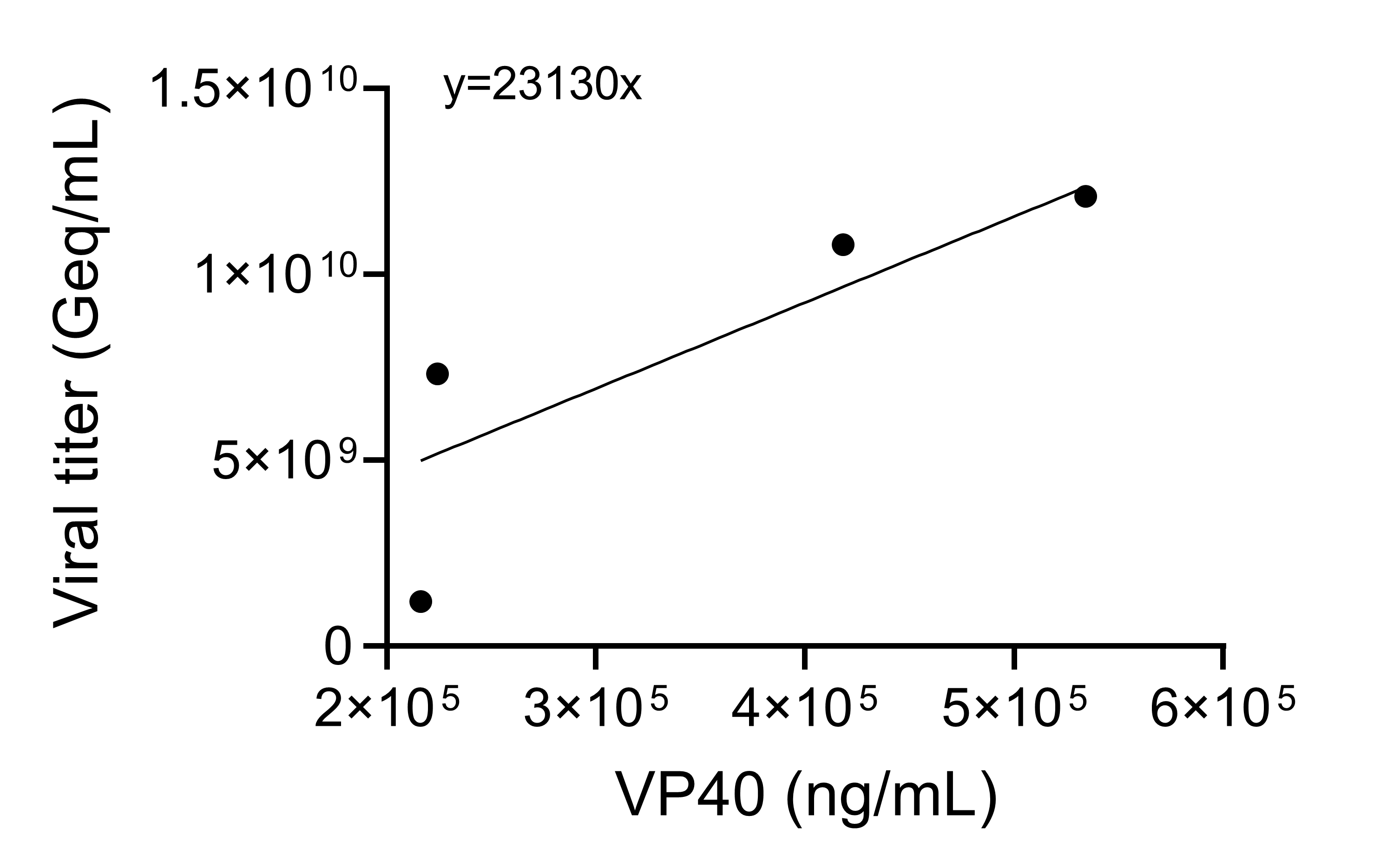

Supplement: S6 Fig — (TIF) [file ppat.1010039.s006.tif]
