# Supplementary material for: Species-specific quantification of circulating ebolavirus burden using VP40-derived peptide variants
Source: PLoS Pathog. 2021 Nov 8;17(11):e1010039. doi: 10.1371/journal.ppat.1010039 (PMC8601621; doi:10.1371/journal.ppat.1010039)
Supplement: S1 Data — (DOCX) [file ppat.1010039.s010.docx]

**S1 Data**

The evaluation of method LOD using MALDI-TOF as the readout was performed using human plasma spiked with recombinant VP40 protein from EBOV (**A** and **B Figs**), SUDV (**C** and **D Figs**) and BDBV (**E** and **F Figs**). To estimate the LOD, we require both peptide 8 and 12 are identified with peak SNR ≥3, and the one with lower SNR defines the assay LOD by comparing its SNR to 3. The average SNRs of EBOV peptide 8 and 12 at 3.125 nM were 13.2 and 19.3 respectively, and the average SNRs of SUDV peptide 8 and 12 at 3.125 nM were 45 and 9.1 respectively. Overall, the LOD for peptide 12 is lower than that of peptide 8 among three species, as indicated by its higher SNR at compared to that of peptide 12. We estimated the assay LOD for EBOV and SUDV as 3.125 nM. This is a conservative estimation, considering the cutoff is 3. However, the peptide peaks with low intensities showed a high variation of SNR, and SNR showed a non-linear relationship with the concentration, making it challenging to accurately calculate the LOD based on SNR. Nonetheless, the two peptides can be simultaneously and unambiguously identified at a concentration not less than 3.125 nM or 110 ng/mL of VP40 from EBOV and SUDV.

For BDBV peptides 8 and 12, the average SNRs at 12.5 nM were 4.6 and 18.9 for peptide 8 and 12. As BDBV VP40 sample contains Tween-80 as preservative (<https://www.sinobiological.com/recombinant-proteins/ebov-ebola-virus-vp40-40448-v07e>), which suppresses the signal of peptide in MALDI-MS, the peak intensity of its peptides after IP-MALDI is lower than those from EBOV or SUDV. EBOV and SUDV VP40 were purchased from another company IBT BioServices, the protein was dissolved in HEPES buffer pH 7.5 containing sodium chloride, 5% glycerol and 0.1% Triton-X. Therefore, the difference in buffer component makes it difficult to compare the peptide peak intensity as well as assay LOD between BDBV and other two species.

As a result, the LOD for BDBV VP40 using MALDI-TOF MS was 12.5 nM. Due to the existence of polymer tween-80 in this sample, the actual LOD was expected to be lower than 12.5 nM.

For BDBV, peptide 12 can be detected with SNR greater than 3 at 3.125 nM, however, the average SNR of peptide 8 was 2.2. This could be explained as the BDBV VP40 protein sample contains polymers that suppresses the signal of targeted peptides.

P12, S/N 19.4

P12, S/N 19.5

P12, S/N 23.2

P8, S/N 25.4

P8, S/N 34.2

P8, S/N 33

**Fig A**. The MALDI-TOF spectra of EBOV VP40 peptide 8 and 12 in human plasma spiked with 12.5 nM recombinant protein. The peptide eluents after IP were spotted in triplicates. The signal-to-noise ratio (S/N) is labeled for each peptide target.

P12, S/N 16.5

P12, S/N 20.4

P12, S/N 20.9

P8, S/N 12.9

P8, S/N 13.1

P8, S/N 13.7

**Fig B**. The MALDI-TOF spectra of EBOV VP40 peptide 8 and 12 in human plasma spiked with 3.125 nM recombinant protein. The peptide eluents after IP were spotted in triplicates. The signal-to-noise ratio (S/N) is labeled for each peptide target.

P12, S/N 121.9

P12, S/N 105.4

P8, S/N 20.3

P8, S/N 19.3

P8, S/N 31.5

P12, S/N 155

**Fig C**. The MALDI-TOF spectra of SUDV VP40 peptide 8 and 12 in human plasma spiked with 12.5 nM recombinant protein. The peptide eluents after IP were spotted in triplicates. The signal-to-noise ratio (S/N) is labeled for each peptide target.

P8, S/N 6.8

P12, S/N 29.8

P8, S/N 9.7

P12, S/N 47.3

P8, S/N 10.9

P12, S/N 58

**Fig D**. The MALDI-TOF spectra of SUDV VP40 peptide 8 and 12 in human plasma spiked with 3.125 nM recombinant protein. The peptide eluents after IP were spotted in triplicates. The signal-to-noise ratio (S/N) is labeled for each peptide target.

**
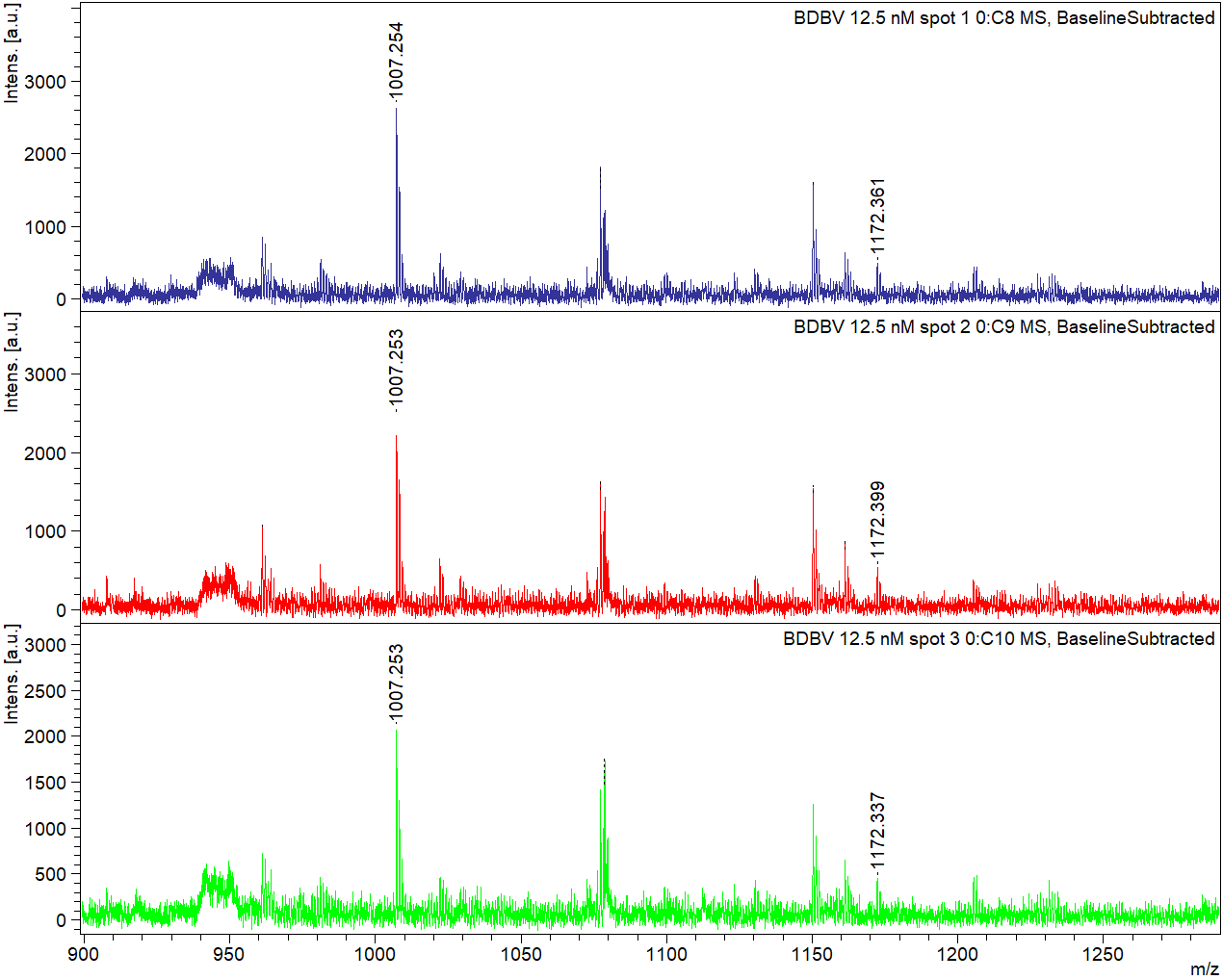
**

P8, S/N 4

P12, S/N 15.7

P8, S/N 4.5

P12, S/N 18.5

P8, S/N 5.2

P12, S/N 22.5

**Fig E**. The MALDI-TOF spectra of BDBV VP40 peptide 8 and 12 in human plasma spiked with 12.5 nM recombinant protein. The peptide eluents after IP were spotted in triplicates. The Signal-to-noise ratio (S/N) is labeled for each peptide target.

P8, S/N 2.2

P8, S/N 2.1

P12, S/N 8.4

P12, S/N 12.6

P12, S/N 16.3

P8, S/N 2.3

**Fig F**. The MALDI-TOF spectra of BDBV VP40 peptide 8 and 12 in human plasma spiked with 3.125 nM recombinant protein. The peptide eluents after IP were spotted in triplicates. The Signal-to-noise ratio (S/N) is labeled for each peptide target.
