# Supplementary material for: Species-specific quantification of circulating ebolavirus burden using VP40-derived peptide variants
Source: PLoS Pathog. 2021 Nov 8;17(11):e1010039. doi: 10.1371/journal.ppat.1010039 (PMC8601621; doi:10.1371/journal.ppat.1010039)
Supplement: Attachment — Submitted filename: response letter.docx [file ppat.1010039.s011.docx]

**Part I - Summary**
Please use this section to discuss strengths/weaknesses of study, novelty/significance, general execution and scholarship.

Reviewer #1: In the manuscript entitled “Species-specific quantification of circulating Ebolavirus burden using VP40-derived peptide variants,” Shu and colleagues develop an immunoprecipitation (IP)-based mass spectrometry (MS) assay to distinguish human-pathogenic ebolaviruses based on amino acid differences in VP40. The authors first identify two immunogenic tryptic peptides that, together, can differentiate between EBOV, SUDV, BDBV, and TAFV based on their amino acid sequences. The authors then use these peptides to produce antibodies, and they incorporate these antibodies into their IP-MS assay. Using this assay the authors were able to distinguish between EBOV, SUDV, and BDBV VP40 in spiked human plasma. The authors were also able to detect EBOV in samples obtained from infected NHPs. Together, these data suggest that this novel IP-MS assay could be used as a diagnostic tool, although its advantages in comparison to standard RT-qPCR diagnostic tests are not clear. Indeed, while this study provides proof of principle, additional work is required to demonstrate whether the IP-MS assay described here will provide any use in a clinical or field setting.

Response: Currently, there are 10 commercial nucleic acid assays for EVD. Seven of them only detect single species and multiple probes are needed to cover all species. In contrast to standard RT-qPCR tests, the IP-MS assay only requires two peptide antibodies to cover and differentiate the four known species that cause human disease. The streamlined sample preparation and detection design of the IP-MS assay can be employed in a laboratory that has minimum biosafety equipment, and its results are less prone to be affected by sample quality, such as PCR inhibitors or nucleic acid degradation during extended sample storage. Mutations at any position in a sequence target by an RT-qPCR test can disrupt performance, but only those mutation that alter amino acid sequence can affect IP-MS assay performance.

This study also acknowledges the need for safe and rapid sample handling, stable reagent storage conditions, and power demands for assays that are performed in field conditions versus clinical settings. It therefore also focuses on optimizing the assay for use in ambient conditions and integrating it with a miniature MS that is portable, and does not require a vacuum pumps or other accessories that that would limit it used in resource limited settings.

Reviewer #2: In “Species-specific Quantification of Circulating Ebolavirus Burden using VP40-derived Peptide Variants”, Shu et al. describe the development of a quantitative assay for EBV using species-specific VP40 peptide variants. The objectives of the study are clearly defined and articulated, with a strong experimental method, excellent figures, and a well written results section. The discussion goes some way to listing the strengths and weaknesses of the data, the latter of which is the absence of any clinical material validation using the methods described. However, the assays parameters are discussed in relation a comparable method (the LFI ReEBOV RDT) and other more standard diagnostic tests (qRT-PCRs) are included which offers the reader an idea of where the newly described technique would stand in a diagnostic setting.

The data presented is very interesting, and shows the potential of Ag-based diagnostics in for these pathogens. I also appreciate the way the authors included thought as to how these assay could be adapted to a field setting which is important for pathogens such as EBV. The main comments I have on the data are that: a) the absence of any data for the remaining EBV species; and b) it is lacking any human clinical samples which would greatly strengthen the data here (although I understand that EBV clinical material is not the easiest to obtain, and the NHP plasma samples represent a suitable surrogate for this).

Response: We agree that our inability of to perform a method validation study using human clinical samples is a major shortcoming of the current manuscript, and regret that we cannot address this issue. However, during the revision, we increased more type of samples for method validation. Specifically, we the thank the author for the suggestion that we should provide data for the remaining ebolavirus species. We have added new data (Fig. 5 and Figs S2-S3) for BDBV, TAFV and RESTV species using inactivated viral material we obtained from BEI Resources since our initial submission.

**Part II – Major Issues: Key Experiments Required for Acceptance**
Please use this section to detail the key new experiments or modifications of existing experiments that should be absolutely required to validate study conclusions.

Generally, there should be no more than 3 such required experiments or major modifications for a "Major Revision" recommendation. If more than 3 experiments are necessary to validate the study conclusions, then you are encouraged to recommend "Reject".

Reviewer #1: Can the authors provide the details for the RT-PCR assay that was used to obtain the data shown in Fig. 5G, H? Were the primers/probe used in this assay validated for diagnostic use? The results in Fig. 5 indicate that the RT-PCR assay is more sensitive than the IP-MS assay, which would make the latter assay less useful as a diagnostic test. Can the authors comment on this discrepancy?

Response: In Fig. 5G, H, the VP40 content was determined by western blotting. SDS-PAGE LDS sample buffer used to inactivate all infected plasma samples, and LDS negatively affected the LoD of the proposed IP-MS assay. The IP-MS assay does not permit sensitive detection of VP40 at Day 4 in animal #7284. We were able to detect the signal at Day 6 in this animal’s plasma, in which the VP40 content was determined as 226 µg/mL. Western blotting also reached its LOD at this post-infection time point. We agree that RT-PCR is currently more sensitive for detecting EBOV infection, however, we believe that advantages of the IP-MS assay with regard to sample handling, process and transportation may make it clinically useful diagnostic test.

The authors position their IP-MS assay as a potential alternative diagnostic test to the standard RT-PCR test. However, based on the description of the IP-MS assay in the Results and Materials and Methods sections, it is not clear whether the IP-MS assay improves upon the “weaknesses” that the authors list on Lines 58-59 for RT-PCR (i.e., cost, time, availability, and operator expertise). Likewise, it is not clear whether the IP-MS is rapid or simple (Line 61). Based on the protocols outlined in the Materials and Methods section, it seems that numerous steps over several hours to days are required to perform the IP-MS, which would make it significantly more complicated than the standard RT-PCR test. Can the authors clarify whether their IP-MS assay is truly a rapid, simple, and inexpensive diagnostic test this, especially in relation to RT-PCR. It would also be helpful to understand how practical the IP-MS assay would be to perform in a resource-limited setting. One suggestion would be to include a flow chart that outlines the steps for IP-MS and RT-PCR tests from clinical sample to results.

Response: We thank the reviewer for this suggestion. We have now added a flow chart that outlines the major steps for the IP-MS and RT-PCR tests as shown in revised Fig. 2 below. We have also added a new section to the Results section titled “Design of an IP-MS assay for VP40 peptide variants” that describes the design of our assay and compare it to well-established RT-PCR assays. The IP-MS assay includes four steps and requires four hours, and is thus comparable to the number of steps and turnaround time of current RT-PCR assays. Notably, the IP-MS assay detects VP40-derived peptides and is less sensitive to inactivation buffers that may impede antibody-antigen recognition in traditional immunoassays. IP-MS also does not employ time consuming reduction/alkylation of disulfide bonds between cysteine residues or a 12-16 hours tryptic digestion step employed in traditional MS-based shotgun proteomic analysis.

Extracted RNA quality is very important for RT-PCR assay performance. RT-PCR assays targeting the Ebola virus glycoprotein (GP) gene, for example, first need to evaluate RNA extraction quality. QIAGEN has created a robust set of internal controls (details available at “https://www.qiagen.com/us/spotlight-pages/newsletters-and-magazines/articles/correct-interpretation-negative-results/”) that allow accurate detection of PCR inhibition and extraction failure. These exogenous heterologous internal controls carry a unique artificial sequence that is not present in any biological sample, and is detected using primers and probes that differ from those specific to the target pathogen. An internal control (IC) DNA or RNA template is spiked into samples during PCR or RT-PCR setup to permit evaluation of whether or not the amplification process occurred without inhibition. An IC template can also be spiked into the sample prior to extraction to additionally control for extraction failure. The IC RNA template additionally controls for successful reverse transcription in RT-PCR assays. If the IC is amplified within manufacturer-designated ranges, further quantitative analysis of the viral target is performed.

**Fig 2. Comparison of the IP-MS assay for VP40 peptide variants with an RT-PCR assay for the GP gene.** Both assays employ four steps for sample analysis. (**A**) In the IP-MS assay for VP40 peptide variants, samples are inactivated using a method described by 2005 CDC Interim Guidance and digested to release the target VP40 peptides, which are then immunoprecipitated, and analyzed by mass spectrometry to detect mass differences among the peptide 8 and 12 sequences encoded by different ebolavirus species. The VP40 p8 and p12 target peptides and internal standard (IS) peptide are captured during immunoprecipitation (IP) by target-specific antibody-coupled magnetic beads. The peak area ratio between known amounts of VP40 p8 (blue peak) and its corresponding IS peptide (purple peak) is used to generate a standard curve to permit quantification of endogenous VP40 in clinical samples. (**B**) In the RT-PCR assay for the GP gene, a carrier RNA and a high concentration internal control are spiked into the sample before extraction. Each extracted RNA sample is tested with the internal control RT-PCR RNA Assay to evaluate the yield of the spiked-in internal control. If the internal control amplified within manufacturer-designated ranges, further quantitative analysis of the viral target is performed using standard procedures.

Can the authors evaluate their IP-MS assay using BDBV and TAFV VLPs as well? Without these data it is difficult to conclude that the assay is capable of differentiating between all human-pathogenic ebolaviruses. Further, if at all possible, the authors should consider evaluating additional samples from infected NHPs (or other animal models) infected with EBOV, SUDV, or BDBV. Additional data points would be extremely helpful in demonstrating that this assay is robust and specific.

Response: We thank the reviewer for the suggestion. We were not able to obtain BDBV and TAFV VLPs, but have now added new data (Fig. 5 and Figs S2-S3) for BDBV, TAFV and RESTV species using inactivated viral material we obtained from BEI Resources since our initial submission.

Reviewer #2:  Given that the nucleotide sequences are published, and the system exist to generate the VLPs (used here), I think data where BDBV and TAFV VLPs were analyzed should be included. I know that some BDBV is included in the supplementary material, but given as it is mentioned in the abstract and introduction, I think it should be included in the main text. However, if TAFV (and potentially RESV and BOMV?) were excluded from the analysis early on (presumably due to lack of fatal disease in humans) then this should be clearly articulated in a sentence early on to avoid confusion.

Response: We thank the reviewer for the suggestion. We were unable to find a vendor that could supply available BDBV and TAFV VLPs, and our enquiry to a vendor able to generate these VLPs indicated that small-scale production of such VLPs, would require 5 to 8 months and cost ~$32,000 per VLP, which was not possible in the time allowed for our response. VLPs employed in this study were also employed to generate a standard curve for VP40 quantitation. We employed p8 for quantification, and p8 sequence varied only in SUDV, so that it should be possible to evaluate the amount of VP40 present using on the two standard curves presented in Figure 4. We have added BDBV and TAFV data in the current Fig. 5. RESTV and BOMV, which are less lethal than the other four species, were excluded because their VP40-derived p8 and p12 sequences were not distinguishable from those of BDBV, as shown in S1. Fig.

**S1 Fig. The species-specificity of three VP40 tryptic peptides and their masses distribution among ebolavirus species.** (**A**) Three VP40 peptide candidates with peptide sequences that exhibit distinct species specificities, where dots indicate species that can be distinguished from unmarked species based on the corresponding peptide. (**B**) Mass differences and amino acid substitutions of the three peptides among the six species. The EBOV peptides were selected as reference sequences for all comparisons, and substitutions from these reference sequences are marked in blue. Sequences used as antigens for antibody generation are marked with asterisks.

• Given that the authors describe the potential diagnostic uses of this technology (which is probably the most novel and exciting aspect of this work) I think more work needs to be carried out in this area. As previously mentioned, human clinical samples would be the idea choice here, but given the difficulty of obtaining these (with detailed matched clinical details) NHP plasma represent a suitable and ideal surrogate. However, the data would be much stronger if greater numbers were analyzed here rather than the 2 animals currently included. The one important question that I think could be answered here to increase manuscript strength would be “how early in infection can VP40 be detected”. This data, along with its variability (this is where I think having more numbers would be very useful) would be extremely useful and, if possible, should be included somewhere in the manuscript.

Response: We thank the reviewer for this suggestion. To evaluate the ability of our assay to detect EVD at the early stage of infection, we endeavored to obtain plasma from NHPs at 3 days post-infection with EBOV, which have been viral loads of 10^5^ to 10^6^ Geq/mL, the mean EBOV levels in patient serum at initial evaluation. However, these samples will not be shipped and available for testing until a week after the submission deadline of this response, which we were not able to extend. We hope to be given the opportunity to include this data in the final version of this manuscript.

**Part III – Minor Issues: Editorial and Data Presentation Modifications**
Please use this section for editorial suggestions as well as relatively minor modifications of existing data that would enhance clarity.

Reviewer #1: Lines 13-14: Reston virus is not likely a human pathogen, whereas Tai Forest virus has caused at least one recorded case of severe disease in a human.

Response: We thank the reviewer for this comment. We corrected the text to now state “Six ebolavirus species are reported to date, including human pathogens Bundibugyo virus (BDBV), Ebola virus (EBOV), Sudan virus (SUDV), and Taï Forest virus (TAFV); non-human pathogen Reston virus (RESTV); and the plausible Bombali virus (BOMV).”

Line 15: Spelling error: “caused”
Response: We corrected this spelling error.

Line 25, 44-45: Filovirus nomenclature in this line should be correct. “EBOV” and “SUDV” refer to the viruses, not the species. Species cannot cause lethal disease, but viruses can.
Response: We thank the reviewer for this critique. We have now altered the text to read “Among them, BDBV, EBOV and SUDV ebolavirus are highly lethal human pathogens. TAFV ebolavirus causes severe but nonlethal human disease. RESTV ebolavirus only caused asymptomatic human infection. The pathogenicity of BOMV ebolavirus for humans is unclear (4).”

Line 26 and elsewhere: As far as this reviewer is aware, “EBV” is not a commonly used or accepted abbreviation for “ebolavirus.” Consider replacing all instances of “EBV” with “ebolavirus.”
Response: We thank the reviewer for this suggestion, and we replaced all instances of “EBV” with “ebolavirus”.

Line 26: The authors state that the proposed assay differentiates four of the six known ebolavirus species, but the manuscript primarily describes assays performed with material derived from EBOV and SUDV, with some assays including material derived from BDBV. Although the proposed IP-MS assay theoretically allows differentiation of TAFV, the authors do not provide these data. Suggest revising this statement accordingly.

Response: We have now added data for generated with gamma-irradiated RESTV and TAFV ebolavirus material and infected Vero-6 cell lysates. These results show that all species tested can be correctly identified by the proposed IP-MS assay based on the species-specific VP40 peptide variants. We believe that this now justifies the original statement, but we can still revise it if the reviewer deems it necessary.

Line 157: Based on Fig 1E, it appears that VP40 p8 is conserved among all human-pathogenic ebolaviruses except for SUDV, not EBOV.

Response: We thank the reviewer for catching this typo, and have corrected EBOV to SUDV.

Line 271: Spelling error: “BDBV”

Response: We have corrected this error.

Line 284: Spelling error: “authentic”

Response: We have corrected this spelling error.

Line 323: Were plasma samples obtained from NHP 436 on Day 7 PI? If so, why was no VP40 signal detected in the Western blot in Fig. 5A?

Response: Animal #436 died on Day 7 PI, and neither a viral titer or VP40 protein could be detected in a post-mortem blood sample drawn from the heart.

Lines 353-357: It is a bit misleading to begin the discussion section with a description of virus isolation as the gold-standard method for confirming ebolavirus infection. While this is true, it does not seem likely that the IP-MS assay will be able to compete with this gold-standard. Rather, the IP-MS assay is, at best, an alternative to RT-PCR assays and, perhaps, RDT assays.

Response: We thank the reviewer for this comment. We have deleted this paragraph in order to make our discussion more relevant to the current need in this field, that is, an RDT enabling quantitative and species-distinguishable detection of VP40.

Lines 383-385: Suggesting re-writing this sentence to avoid confusion.

Response: We have revised this sentence to read “The LOD of the ReEBOV RDT was determined to be 4.7 ng/test in serum and 9.4 ng/test in whole blood, equivalent to 3.0×10^5^genomes/mL. In comparison, the LOD of our newly developed IP-MS assay was 2.8 ng/test and 0.7 ng/test in plasma for EBOV and SUDV VP40, respectively, when analyzed by MRM, equivalent to 4.6×10^5^ genomes/mL.” We believe this should remove the confusion caused by using two concentration units. We also replaced the genomes equivalence range to instead indicate only the lowest value in order to highlight the assay LOD.

Lines 407-409: The authors seem to suggest that their IP-MS assay could be used to identify VP40 mutations. While this may be true specifically for the single mutation in p8 and the three mutations in p12 that are assessed in this study, it would not necessarily be true for other mutations within these peptides, would it? In any case, it does not seem practical to suggest that IP-MS be used to define protein mutations when gene sequencing can be performed easily, including in the field.

Response: We thank the reviewer for this comment. We agree with the reviewer that gene sequencing is the most convenient way to identify protein mutations. We don’t intend to suggest using the proposed IP-MS assay be used to identify unknown mutations. However, once such mutations are identified through gene sequencing, we suggest the proposed assay could be used to identify known VP40 sequence mutations by selecting peptide targets that cover these mutation sites, although this requires generation of new peptide antibodies specific for these regions. Such mutations may affect antibody binding affinity among variants, as we observed in this study. However, these results suggest that a 3- to 30-fold difference in affinity should not affect the successful identification of both variants. Further is may be possible to minimize such affinity differences by omitting mutation sites from the sequence region used to generate the peptide-specific antibody for this target, as exemplified by the p12 antibody.

In theory, MS identification and quantification of target peptides can be achieved by two strategies. One uses an untargeted approach where a full mass scan is followed by data-dependent acquisition of tandem MS spectra of intense peptide peaks. The other employs a targeted approach, such as MRM-MS, where the MS method to analyze a peptide sequence is pre-determined and would thus miss unknown mutations in a target peptide sequence. Nevertheless, an untargeted strategy permits the identification of all peptides captured by immunoprecipitation. By considering potential amino acid substitutions during MS/MS spectral annotation, this strategy could identify novel mutations in VP40 target peptides. However, this is out of the scope of this work, and we therefore revised the paragraph to avoid confusion.

Reviewer #2:

• Line 44: Expand EBV

Response: We thank the reviewer for this suggestion and have replaced EBV with ebolavirus as mentioned above.

• Line 157: Should be SUDV rather than EBOV I think?

Response: We have now corrected EBOV to SUDV in this sentence.

• Line 207: glycoprotein should not be capitalized

Response: We have now corrected this error.

• Line 321: Include the type of serial dilution (2-fold I think?) here.

Response: We now indicate that this is a two-fold serial dilution in the corresponding text.

• Line 359-362: Line discussing the types of inactivation should have a reference (or multiple references).

Response: We thank the reviewer for this suggestion and have added four references here.

• Line 431: Given their importance in this work, in this section I think the specifics of how the VLPs were generated, purified, and quantified should be expended on rather than refereeing to a single reference.

Response: We have added the details of VLP generation to the Methods section. VLPs were manufactured by Paragon Bioservices and produced by transfecting HEK293F cells with Ebola Zaire virus GP and VP40 genes in pWRG expression vectors, essentially as previously described in Swenson et al 2004. Full-length cDNAs encoding ebolavirus (species Zaire, EBOV) VP40 or GP were cloned into the mammalian expression vector pWRG7077. 293T cells were transfected with plasmids using the lipofectamine reagent (Invitrogen, Carlsbad, CA, USA) according to the manufacturer’s instructions. Transfected cells were incubated at 37°C under 5% CO2 for 48 h prior to harvesting VLPs. VLPs were prepared essentially as previously described (J. Exp. Med., 195 (2002), pp. 593-602). Briefly, supernatants were collected 48 h after transfection, overlaid on 20% sucrose and ultracentrifuged at 26 000 rpm for 2 h. Pelleted material was further purified by a sucrose step gradient consisting of 60%, 40% and 10% layers. After ultracentrifugation, the VLPs were recovered at the interface of 10/40% and 40/60% layers.

• Line 464: Age, sex, and route of inoculation for the NHPs should be included.

Response: We now include information on the animal’s age, sex and route of inoculation in the current version of Table S3.

• Line 533: Missing a VP40 after the SUDV I think?

Response: We thank the reviewer for this observation, and have added the missing “VP40” in Line 575.

• Figure 1D: No reference in the legend to what the blue and purple (closed an open) dot represent, or what the dotted blue line represents.

Response: We have added the details of these labels in the legend as “Blue and purple dots respectively indicate data for the EBOV and SUDV species, while the closed or open dots indicate the antigenicity scores for a given peptide sequence that are above (closed) or below (open) the antigenicity score threshold (dotted red line) of a good antigen peptide defined by each algorithm.”

• Figure 3A: Several artifacts present, looks like from masking boxes. Also, this makes it look as if the VP40 and GP genes are expressed as a single CDS? If the whole plasmid is represented here then each (relevant) CDS should be individually displayed. It would be useful to show what the arrows represent (presumably transfection and VLP release) as well.

Response: We thank the reviewer for this technical question and suggestion. After reviewing the previous publication and the contract with Paragon Bioservices (Baltimore, MD), we determined that GP and VP40 were separately inserted into the pWRG7077 plasmids, and 293T cells were co-transfected with these two plasmids to express both genes and assemble them into VLPs. We therefore revised Fig. 3A as shown below.

Revised Fig. 3

• Figure 3B: What do the wells represent? If they are dilution of the VP40 then this should be indicated on the figure. A protein weight marker would also be relevant here.

Response: We have now numbered and defined their contents in the figure legend now. Wells 1-5 and 6-10 respectively indicate a two-fold serial dilutions of recombinant VP40 protein (starting from 0.87 µg of VP40) and VLP protein extract (starting from 2.5 µg total VLP protein). The position of a 38 kDa protein marker that migrates close to VP40 is indicated next to each Western blot.

• Figure 4C: this is not referred to in the main text.

Response: We thank the reviewer for this comment, and now refer this figure (now Fig. 5C due to the addition of a figure) in Line 317.

• Figure 5A and B: Again, please indicate what each of the wells contains, and include a protein size marker if possible.

Response: We thank the reviewer for this comment, and have added these details in the legend as follows: “Wells 1-5 represent a two-fold serial dilution of recombinant VP40 (starting from 870 ng VP40). Wells 6-10 represent a two-fold dilution series of plasma form an EBOV-infected NHP (starting form undiluted plasma), where 2μL of diluted and undiluted plasma were loaded into each well. The VP40 band is indicated by a black arrowhead.“ We did not add a protein marker to the gels in Fig. 5A and B. We instead directly used recombinant VP40 as a marker, since it was observed as a single band upon SDS-PAGE in Fig. 3B.

• Figure 5G and H: Error bars for the RT-PCR data (red bars)?

Response: We have added these error bars to the revised Fig. 6 shown below. The raw data for RNA quantity was also provided in Table S3 in the revised manuscript.

Revised Fig. 6

• Supporting info S6 data: should be BDBV in the title?

Response: We thank the reviewer for this comment, and corrected the title as “The MALDI-TOF spectra of BDBV VP40 peptide 8 and 12 in human plasma spiked with 3.125 nM recombinant protein. The peptide eluents after IP were spotted in triplicates. The signal-to-noise ratio (S/N) is labeled for each peptide target.”
